# The Role of Pre-therapeutic ^18^F-FDG PET/CT in Pediatric Hemophagocytic Lymphohistiocytosis With Epstein-Barr Virus Infection

**DOI:** 10.3389/fmed.2021.836438

**Published:** 2022-01-21

**Authors:** Xia Lu, Ang Wei, Xu Yang, Jun Liu, Siqi Li, Ying Kan, Wei Wang, Tianyou Wang, Rui Zhang, Jigang Yang

**Affiliations:** ^1^Nuclear Medicine Department, Beijing Friendship Hospital Affiliated to Capital Medical University, Beijing, China; ^2^National Center for Children's Health, Hematology Center, Beijing Children's Hospital, Capital Medical University, Beijing, China

**Keywords:** hemophagocytic lymphohistiocytosis (HLH), Epstein-Barr virus (EBV), ^18^F-FDG PET/CT, differential diagnosis, prognosis, children

## Abstract

**Objective:**

To evaluate the role of pre-therapeutic ^18^F-FDG PET/CT in pediatric hemophagocytic lymphohistiocytosis (HLH) with Epstein-Barr virus (EBV) infection.

**Methods:**

This retrospective study included 29 HLH children (1–16 years) with EBV infection, who underwent pre-therapeutic ^18^F-FDG PET/CT from July 2018 to November 2020. Pathology results were considered as the reference standard. These patients were divided into two groups: EBV-induced malignancy-associated HLH (M-HLH, *N* = 9) and EBV-induced non-malignancy-associated HLH (NM-HLH, *N* = 20). The regions of interest (ROIs) of the liver, spleen (Sp), bone marrow (BM), lymph nodes (LN), hypermetabolic lesions, liver background (LiBG), and mediastinum (M) were drawn with software 3D-Slicer. The volumetric and metabolic parameters, including maximum standard uptake value (SUV_max_), metabolic tumor volume, and total lesion glycolysis of these ROIs, clinical parameters, and laboratory parameters were compared between the two groups. The efficiency of the above parameters in predicting the treatment response and overall survival (OS) was analyzed.

**Results:**

Receiver operating characteristic curve analysis indicated that SUV_max_-lesions and SUV_max_-LN/M (AUC = 0.822, 0.819, cut-off = 6.04, 5.74, respectively) performed better in differentiating M-HLH from NM-HLH. It had the best diagnostic performance when age was added with the SUV_max_-LN/M (AUC = 0.933, sensitivity = 100%, specificity = 85.0%). The presence of extranodal hypermetabolic lesions in multiple organs indicated the M-HLH (*P* = 0.022). Older age, higher SUV_max_-LN and SUV_max_-lesions, and the presence of serous effusion were associated with poorer treatment response at the 2nd and 4th week (not reaching partial remission). Multivariate analysis showed that SUV_max_-lesions > 7.66 and SUV_max_-Sp/LiBG > 2.01 were independent prognostic factors for overall survival (*P* = 0.025, 0.036, respectively).

**Conclusions:**

^18^F-FDG PET/CT could be a valuable technique for identifying the underlying malignancy and predicting prognosis in pediatric HLH with EBV infection. M-HLH could be considered when SUV_max_-lesions > 6.04, SUV_max_-LN/M > 5.74, and the presence of extranodal hypermetabolic lesions in multiple organs on ^18^F-FDG PET/CT. SUV_max_-lesions and SUV_max_-Sp/LiBG might be independent prognostic factors for OS.

## Introduction

Hemophagocytic lymphohistiocytosis (HLH) is a clinical syndrome of uncontrolled activation of the immune system due to a variety of reasons, charactering as excessive elevated inflammatory cytokines and multiple organs damages (1). As a rare disease with poor outcomes, HLH have an estimated yearly incidence of one to ten per million children and a five-year survival rate of 54% (2, 3). HLH can be classified into two forms, primary HLH with related gene mutations, and secondary HLH associated with infection, malignancy, or autoimmune disorders (4).

The incidence of HLH with Epstein-Barr virus infection is especially high in Asia (5). In the background of EBV infection, HLH can be driven by pure EBV infection (abbreviated as EBV-HLH in the paper), chronic active EBV infection [CAEBV, which has been defined by the World Health Organization classification as lymphoproliferative disorders (LPD)], and lymphoma (6). LPD can be divided into grade 1–3, corresponding to category A1–A3 classified by Ohshima et al. (7). The LPD grade 3 is considered as malignant disorders (8). Patients with EBV-induced malignancy-associated HLH (abbreviated as M-HLH in the study, including LPD grade 3 and lymphoma) have poorer prognosis than EBV-induced non-malignancy-associated HLH (abbreviated as NM-HLH, including LPD grade 1–2 and EBV-HLH). It is reported that higher pathologic grade predicts poorer prognosis (9). M-HLH patients would die of fulminant disease progression without intensive treatment, such as aggressive immune suppression, chemotherapy, and allogeneic hematopoietic stem cell transplant (8). However, it is difficult to differentiate M-HLH from NM-HLH, owing to the high overlap of clinical manifestation.

^18^F-FDG PET/CT is a whole-body scan widely used in many diseases, such as infection, malignant disease, and rheumatic immunity disease (10). In varieties of lymphoma, it is used in differential diagnosis, treatment decision, response evaluation and prognosis prediction (10, 11). ^18^F-FDG PET/CT is better than conventional radiography, as it can display the extranodal lesions better and measure the metabolism in semi-quantitative analysis (12). ^18^F-FDG PET/CT could evaluate the involved organs of potential disease in HLH and guide the biopsy of lesions (13). In HLH with EBV infection, ^18^F-FDG PET/CT is now recommended for the detection of neoplastic lesions, as the lymph nodes and extranodal organs are usually involved, especially in M-HLH (6). Moreover, studies reported that PET/CT parameters, such as SUV_max_ spleen/mediastinum ratio, could predict prognosis of HLH (14).

We speculate that ^18^F-FDG PET/CT plays a certain role in detecting the potential malignancy and predicting prognosis in pediatric HLH with EBV infection, which is not investigated by now. Therefore, this study mainly included two aspects, to differentiate M-HLH from NM-HLH, and to predict the treatment response and overall survival (OS) by ^18^F-FDG PET/CT.

## Materials and Methods

### Patients

**Figure 1:**
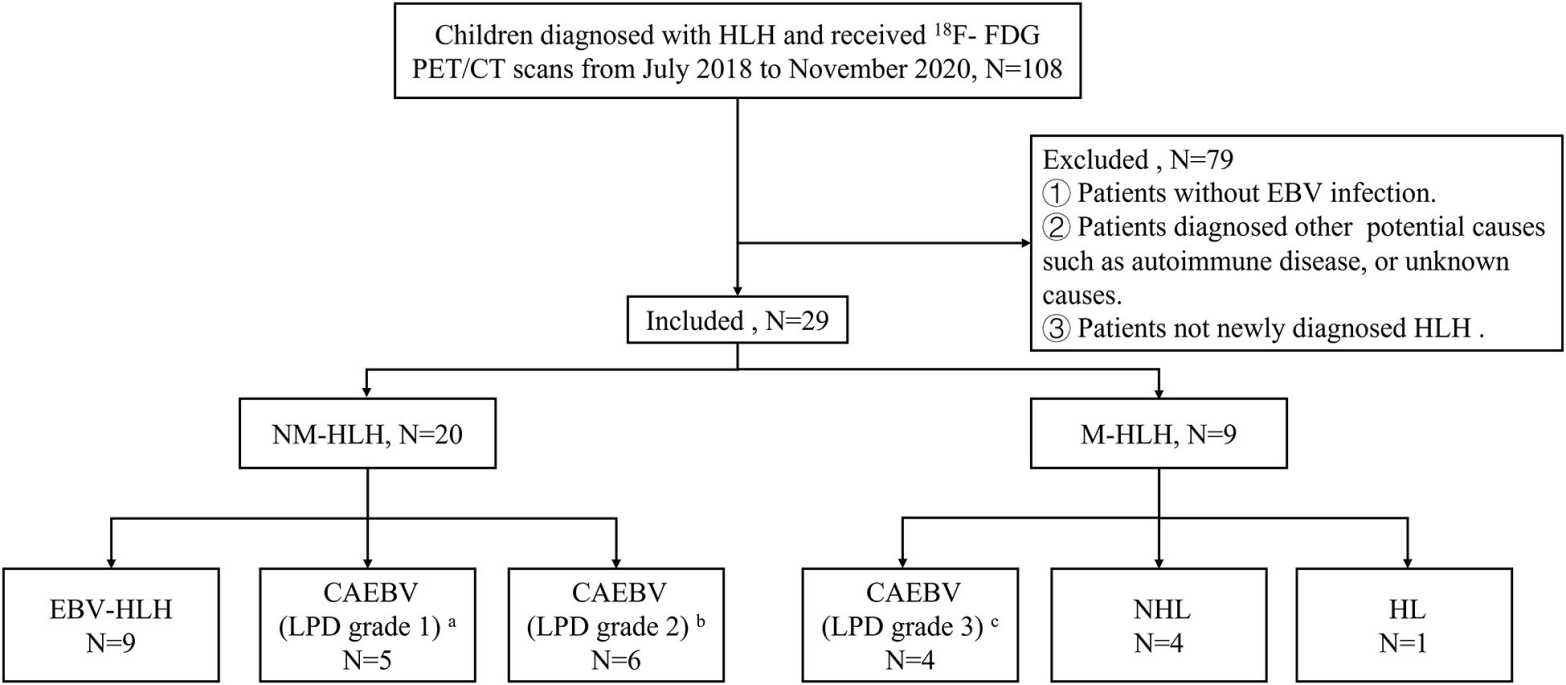
Flow diagrams of study populations. a: category A1, b: category A2, c: category A3, in classification with the pathological categories proposed by Ohshima et al. (7). HLH, hemophagocytic lymphohistiocytosis; EBV, Epstein-Barr virus; M-HLH, EBV-induced malignancy-associated HLH; NM-HLH, EBV-induced non-malignancy-associated HLH; EBV-HLH, HLH driven by pure EBV infection; CAEBV, chronic active EBV infection; LPD, lymphoproliferative disorders; HL, Hodgkin's Lymphoma; NHL, non-Hodgkin's lymphoma.

A total of 29 children (≤ 16 years old) were analyzed retrospectively. They were all newly diagnosed HLH with EBV infection and received pre-therapeutic ^18^F-FDG PET/CT from July 2018 to November 2020. All patients met the diagnostic criteria of HLH-2004 protocol and the criteria of EBV infection (15, 16), and the latter included serological evidence of acute or active EBV infection, evidence of EBV DNA in the blood and/or EBER positive in the tissue. Patients were excluded if they had received clinical therapy, including chemotherapy, targeted therapies, or corticosteroid before the scans, or colony-stimulating factor therapy within one week. In all enrolled patients, the potential causes of HLH were diagnosed according to the pathologic findings of lymph nodes, bone marrow or lesions. Then according to the pathology results, patients were divided into two groups, M-HLH and NM-HLH. The patients were followed up until October 2021 by telephone or medical records. OS was calculated from the date of the scan to the date of death for any cause or to the date of last follow-up. The institutional ethics committee of Beijing Friendship Hospital, Capital Medical University, approved the retrospective study and waived the requirement for written informed consent (Figure 1).

### ^18^F-FDG PET/CT Acquisition

All ^18^F**-**FDG PET/CT scans were performed with a Siemens mCT PET/CT scanner (Siemens Medical Solution, Erlangen, Germany). Patients were fasted for more than 6 h, and blood glucose level was controlled at <7.1 mmol/L, before the intravenous administration of ^18^F-FDG (3.7 MBq/kg). A scan from the head to the mid-thigh was performed 60 min after injection. The CT scan was obtained at 120 kV, 200 mA and 3 mm thickness. Then PET was obtained in 3-dimensional mode at 2 min/bed. The CT-based, attenuation-corrected PET images were reconstructed with an iterative algorithm.

### Image Analysis

^18^F-FDG PET/CT images were analyzed by two experienced nuclear medicine physicians on 3D-Slicer (A free and open-source software, widely used by physicians and researchers). The whole liver, spleen and lumbar 1–5 vertebra were drawn as regions of interest (ROIs) of the liver (Li), spleen (Sp), bone marrow (BM), respectively. ROIs of lymph nodes (LN) were drawn on all lymph nodes with short diameter > 0.5 cm. ROIs of lesions were drawn in all the lymph nodes and extranodal lesions. And the extranodal lesions were defined as lesions with higher FDG uptake than the background tissue in extranodal organs, excluding physiological uptake. The SUV_max_ of the liver background (LiBG) and mediastinum (M) were determined by a 3-cm spheric ROI in the liver and a circular ROI drawn within the walls of the aortic arch, respectively. Then metabolic and volumetric parameters, including maximum standard uptake value (SUV_max_), metabolic tumor volume (MTV) and total lesion glycolysis (TLG) of these ROIs, were calculated by the software. The volumetric parameters of liver and spleen were corrected by body surface area [Du Bois (17)] and were abbreviated as MTVc and TLGc. Hypermetabolic LN, Li, Sp or BM were defined when their SUV_max_ > 2.5 or SUV_max_ > SUV_max_-LiBG. Hepatomegaly and splenomegaly were considered when the inferior margin over the costal margin. Lymphadenopathy was considered when short-axis diameter more than 15 mm for Level II and 10 mm for others (18).

### Clinical Information Review

All clinical information was reviewed from medical records, including age, gender, and laboratory results. The laboratory results were obtained within one week before the PET/CT scan, including blood routines (ANC, Hb, PLT), blood biochemical results (albumin, fibrinogen, TG, ALT, AST, LDH), serum cytokine levels (IFN-γ, TNF-α, IL-6, IL-10), serum ferritin, soluble CD25 (sCD25), NK cell activity, EBV DNA copies, ESR, and CRP.

The treatment response of HLH was assessed according to the evaluation criteria proposed by Midwest Collaboration Group of the United States and was divided into three categories: complete remission, partial remission, and no remission (19). The evaluation depended on the following quantified symptoms and laboratory markers of HLH, including levels of sCD25, serum ferritin, blood cell counts (ANC, Hb, PLT), TG, ALT, presence of hemophagocytosis in pathology specimens, and level of consciousness (in patients with central nervous system involvement).

### Statistical Analysis

Data were analyzed with SPSS 26.0 (IBM, Armonk, USA) and figures were generated using GraphPad Prism 8 (GraphPad Software, San Diego, CA). Continuous variables with a skewed distribution were presented as median (range). Categorical variables were presented as numbers [percentages (%)]. Continuous variables were compared using the Mann-Whitney U test, and categorical variables were analyzed with the two-sided Fisher's exact test. Spearman correlation analysis was used for correlation analysis. The positive or negative relationship was interpretated as very high, high, moderate, low, or negligible (|r| = 0.90–1.00, 0.70–0.89, 0.50–0.69, 0.25–0.49, 0–0.24, respectively). Receiver operating characteristic (ROC) curves were calculated to determine the optimal cut-off value. In univariate analysis of OS, the Kaplan-Meier method and log-rank test were used. The Cox proportional hazards model was used for the multivariate analysis of OS. In univariate analysis, *P* < 0.02 was considered statistically significant, and *P* < 0.05 was considered statistically significant in other condition. ^#^*P* < 0.02, ^*^P < 0.05, ^**^P < 0.01.

## Results

### Patients' Characteristics

A total of 29 children aged 1–16 years (median age, 7 years, male: female = 1:1.1) were enrolled in this study. All children were diagnosed by pathological examination. There were 20 cases in NM-HLH group (including nine cases of EBV-HLH and 11 cases of LPD grade 1–2) and nine cases in M-HLH group (including four cases of LPD grade 3 and 5 cases of lymphoma) (Figure 1). The general information and laboratory parameters are listed in Table 1.

**Table 1:** Characteristics of patients and comparison of clinical and ^18^F-FDG PET/CT findings between M-HLH and NM-HLH.

**Parameters**	**Total (***N*** = 29)**	**M-HLH (***N*** = 9)**	**NM-HLH (***N*** = 20)**	***P*** **-value**
**General information**				
Age (median, range)	7 (1–16)	11 (6–16)	3.5 (1–13)	0.004**
Gender (male)	14 (48.3)	4 (44.4)	10 (50.0)	1.000
**Laboratory parameters (median, range)**				
ANC (10^9^/L)	0.91 (0.27–4.52)	1.08 (0.27–3.29)	0.91(0.28–4.52)	0.444
Hb (g/L)	96 (62–128)	107 (62–121)	95 (64–128)	0.627
PLT (10^9^/L)	111 (19–211)	91 (19–201)	126 (30–211)	0.216
Fibrinogen (g/L)	1.51 (1.55–4.07)	1.62 (0.55–4.07)	1.50 (0.80–2.74)	0.982
Serum ferritin (ng/mL)	355.1 (13.2–139139.0)	317.1 (95.8–14717.6)	407.1 (13.2–139139.0)	0.945
TG (mmol/L)	2.49 (1.01–6.48)	2.86 (1.97–5.62)	2.27 (1.01–6.48)	0.183
sCD25(pg/mL)	22982 (165–218875)	36035 (5354–44000)	20169.5 (165–218875)	0.532
NK (%)	15.72 (7.21–23.83)	15.72 (7.21–19.15)	15.695 (11.54–23.83)	0.390
EBV-DNA (whole blood) (x10^5^Copies/mL)	24.6 (0.0155–225)	8.1 (0.0266–109)	33.1 (0.0155–225)	0.472
EBV-DNA (plasma) (x10^5^Copies/mL)	0.411 (0.005–72.8)	0.0597 (0.005–18.4)	0.0295 (0.005–72.8)	0.253
IFN-γ (pg/mL)	40.92 (2.04–625.000)	57.18 (2.04–458.51)	22.23 (2.82–625.00)	0.390
TNF-α (pg/mL)	1.78 (0.00–34.85)	1.96 (0.00–22.70)	1.21 (0.00–34.85)	0.627
IL-6 (pg/mL)	19.78 (1.80–313.98)	27.47 (1.80–128.93)	14.60 (4.37–313.98)	0.871
IL-10 (pg/mL)	22.57 (2.15–827.66)	39.40 (2.15–827.66)	21.62 (5.38–485.45)	0.908
ALT (U/L)	69.4 (5.2–439.8)	108.1 (5.2–375.3)	67.2 (11.6–439.8)	0.694
AST (U/L)	152.6 (13.2–1161.9)	236.2 (13.2–494.0)	137.1 (32.3–1161.9)	0.365
LDH (U/L)	592 (257–7527)	581 (414–3110)	605.5 (257–7527)	0.764
Albumin (g/L)	35.1 (20.0–44.1)	35.9 (27.2–39.8)	34.65 (20.0–44.1)	0.982
ESR (mm/h)	13 (2–88)	13 (2–34)	11.5 (2–88)	0.660
CRP (mg/L)	5 (5–77)	5 (5–77)	5 (5–55)	0.945
**PET/CT general findings (cases, %)**				
Hepatomegaly	29 (100.0)	9 (100.0)	20 (100)	1.000
Hypermetabolic Li	5 (17.2)	3 (33.3)	2 (10.0)	0.287
Splenomegaly	28 (96.6)	9 (100.0)	19 (95.0)	1.000
Hypermetabolic Sp	20 (69.0)	5 (55.6)	15 (75.0)	0.396
Lymphadenopathy	14 (16.6)	5 (55.6)	9 (45.0)	0.700
Hypermetabolic LN	25 (6.2)	9 (100.0)	16 (80.0)	0.280
Hypermetabolic BM	22 (75.9)	7 (77.8)	15 (75.0)	1.000
Serous effusion	18 (62.1)	6 (66.7)	12 (60.0)	1.000
Extranodal lesions	12 (41.4)	6 (66.7)	6 (30.0)	0.074
Extranodal lesions in multiple organs	5 (17.2)	4 (44.4)	1 (5.0)	0.022;*
**Metabolic parameters (median, range)**				
SUV_max_-lesions	3.49 (1.10–19.91)	8.30 (2.48–16.03)	3.10 (1.10–19.91)	0.005**
SUV_max_-M	1.04 (0.059–1.41)	1.04 (0.059–1.34)	1.02 (0.65–1.41)	/
SUV_max_-LiBG	1.63 (0.94–5.00)	1.80 (1.36–5.00)	1.44 (0.94–3.14)	/
SUV_max_-LN	3.11 (1.10–16.03)	7.55 (2.48–16.03)	2.535 (1.10–19.91)	0.004**
SUV_max_-LN/M	3.42 (1.2–16.7)	6.68 (2.4–16.7)	2.97 (1.2–14.6)	0.005**
SUV_max_-LN/LiBG	1.84 (0.81–9.83)	3.59 (1.39–9.83)	1.80 (0.81–9.30)	0.055
MTV-LN (mL)	12.16 (2.69–151.3)	28.71 (3.83–57.62)	9.325 (2.69–151.3)	0.018;*
TLG-LN (mL)	16.37 (2.02–777.62)	83.31 (6.83–213.30)	12.72 (2.02–777.62)	0.015;*
SUV_max_-Li	2.05 (1.29–12.46)	2.34 (1.73–12.46)	1.775 (1.29–6.15)	0.069
SUV_max_-Li/M	2.32 (0.93–16.31)	2.44 (1.76–16.31)	2.165 (0.93–4.52)	0.317
MTVc-Li (mL)	942.10 (568.42–1590.43)	1113.97 (686.68–1590.43)	932.385 (568.42–1482.97)	0.274
TLGc-Li (mL)	1268.14 (648.65–4947.97)	1711.83 (987.11–4947.97)	1201.855 (648.65–2224.83)	0.069
SUV_max_-Sp	2.21 (1.27–8.02)	3.27 (1.50–8.02)	2.155 (1.27–4.55)	0.234
SUV_max_-Sp/M	2.32 (0.91–13.59)	3.41 (1.29–13.59)	2.17 (0.91–4.1)	0.234
SUV_max_-Sp/ LiBG	1.43 (0.81–3.19)	1.60 (0.81–3.19)	1.415 (1.00–2.45)	0.729
MTVc-Sp (mL)	407.9 (144.35–1392.31)	659.29 (351.51–1392.31)	354.295 (144.35–1534.1)	0.011;*
TLGc-Sp (mL)	661.44 (188.63–3614.05)	1307.57 (394.32–3614.05)	529.99 (188.63–2784.64)	0.011;*
SUV_max_-BM	3.39 (1.78–9.02)	3.60 (2.03–9.02)	3.22 (1.78–8.53)	0.167
SUV_max_-BM/M	3.86 (1.28–15.28)	3.89 (1.86–15.28)	3.82 (1.28–6.27)	0.694
SUVmax-BM/LiBG	2.05 (1.10–4.25)	1.80 (1.10–4.04)	2.06 (1.31–4.25)	0.444

*HLH, hemophagocytic lymphohistiocytosis; EBV, Epstein-Barr virus; M-HLH, EBV-induced malignancy-associated HLH; NM-HLH, EBV-induced non-malignancy-associated HLH; EBV-HLH, HLH driven by pure EBV infection; CAEBV, chronic active EBV infection; LPD, lymphoproliferative disorders; HL, Hodgkin's Lymphoma; NHL, non-Hodgkin's lymphoma; ANC, absolute neutrophil count; Hb, hemoglobin; PLT, platelet; TG, triglyceride; sCD25, soluble CD25; NK, natural killer; IFN-γ, interferon-γ; TNF-α, tumor necrosis factor-α, IL: interleukin; ALT, alanine transaminase; AST, aspartate aminotransferase; LDH, lactate dehydrogenase; ESR, Erythrocyte Sedimentation Rate; CRP, C-reactive protein; Li, liver; Sp, spleen; BM, bone marrow; LN, lymph nodes; LiBG, liver background; M, mediastinum; SUV_max_, maximum standard uptake value; MTV, metabolic tumor volume; TLG, total lesion glycolysis; c, corrected*.

* *P < 0.05*,

** *P < 0.01*.

### Diagnostic Performance of ^18^F-FDG PET/CT for Detecting M-HLH

**Figure 2:**
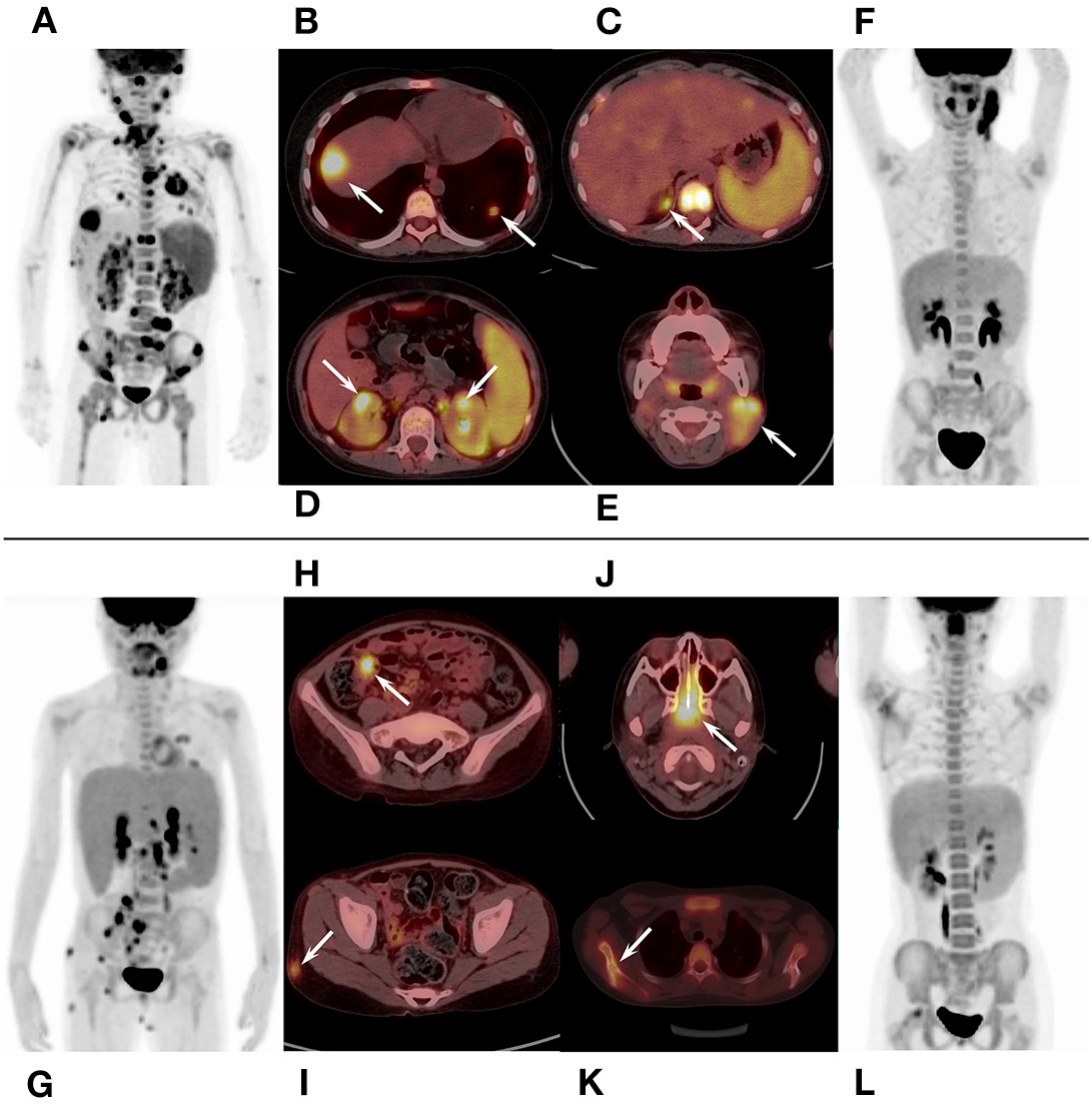
^18^F-FDG PET/CT findings of M-HLH. **(A–D)** A 6-year-old boy with HL, had multiple enlarged hypermetabolic lymph nodes and extronodal hypermetabolic lesions in multiple organs (nasal mucosa, liver, lung, adrenal gland, kidney, and bone marrow). **(E,F)** A 6-year-old boy with LPD grade 3, had local mass of fused lymph nodes in the neck; **(G–I)**, a 10-year-old girl with NHL, had multiple enlarged hypermetabolic lymph nodes and extronodal hypermetabolic lesions in terminal ileum and skin. **(J–L)** A 10-year-old girl with NHL, had hypermetabolic lesions in nasopharynx and multiple lesions in scapula and femur. HLH, hemophagocytic lymphohistiocytosis; M-HLH, EBV-induced malignancy-associated HLH; LPD, lymphoproliferative disorders; HL, Hodgkin's Lymphoma; NHL, non-Hodgkin's lymphoma.

**Figure 3:**
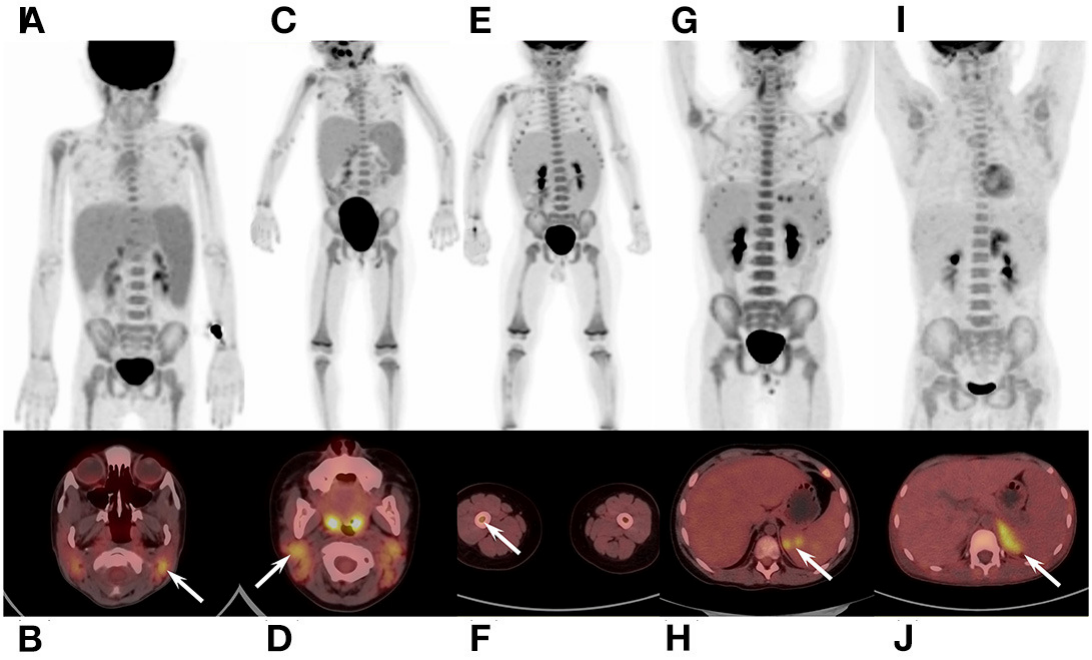
^18^F-FDG PET/CT findings of NM-HLH. **(A,B)** A 5-year-old girl with EBV-HLH. **(C,D)** A 3-year-old girl with LPD grade 2. **(E,F)** a 2-year-old boy with LPD grade 1. **(G,H)** A 12-year-old boy with LPD grade 2. **(I,J)** A 11-year-old boy with LPD grade 2. NM-HLH patients usually show multiple lymph nodes with slightly or moderate increased FDG uptake, in bilateral cervical, mediastinal, axillary, retroperitoneal, pelvic and/or inguinal regions **(A–D,E,G,J)**, without extranodal organ involvement **(A,C)**. Sometimes, however, NM-HLH could show extranodal lesions in single organ, such as bone marrow **(F)**, spleen **(H)**, adrenal gland **(J)**, or muscles. HLH, hemophagocytic lymphohistiocytosis; EBV, Epstein-Barr virus; NM-HLH, EBV-induced non-malignancy-associated HLH; EBV-HLH, HLH driven by pure EBV infection; LPD, lymphoproliferative disorders.

In qualitative and visual analysis, almost all M-HLH children had lymphoma-like presentation in ^18^F-FDG PET/CT, including multiple enlarged lymph nodes with obviously increased FDG uptake, local mass of fused lymph nodes, and/or extronodal lesions, etc. (Figure 2). The presence of extranodal hypermetabolic lesions is helpful for differentiating M-HLH from NM-HLH (*P* = 0.074), and the involvement of multiple organs had better diagnostic performance (*P* = 0.022) (Table 1). The affected organs included spleen, liver, bone marrow, brain, lung, intestine, kidney, adrenal gland, skin, nasal mucosa, muscle, etc. Five of the six patients of NM-HLH with extranodal lesions had only single organ involvement, including bone marrow involvement in one patient with EBV-HLH, spleen, bone marrow, adrenal gland, or muscles involvement in four patients with LPD grade 1–2 (Figure 3).

The lymph nodes and lesions of M-HLH had higher FDG uptake than NM-HLH based on visual analysis (Figures 2, 3). For example, two patients diagnosed as NHL, LPD grade 3, presented as multiple normal-size lymph nodes with obviously increased FDG uptake. And almost all the NM-HLH patients with enlarged lymph nodes (8/9), mostly LPD grade 1–2, had lower FDG uptake than M-HLH.

**Figure 4:**
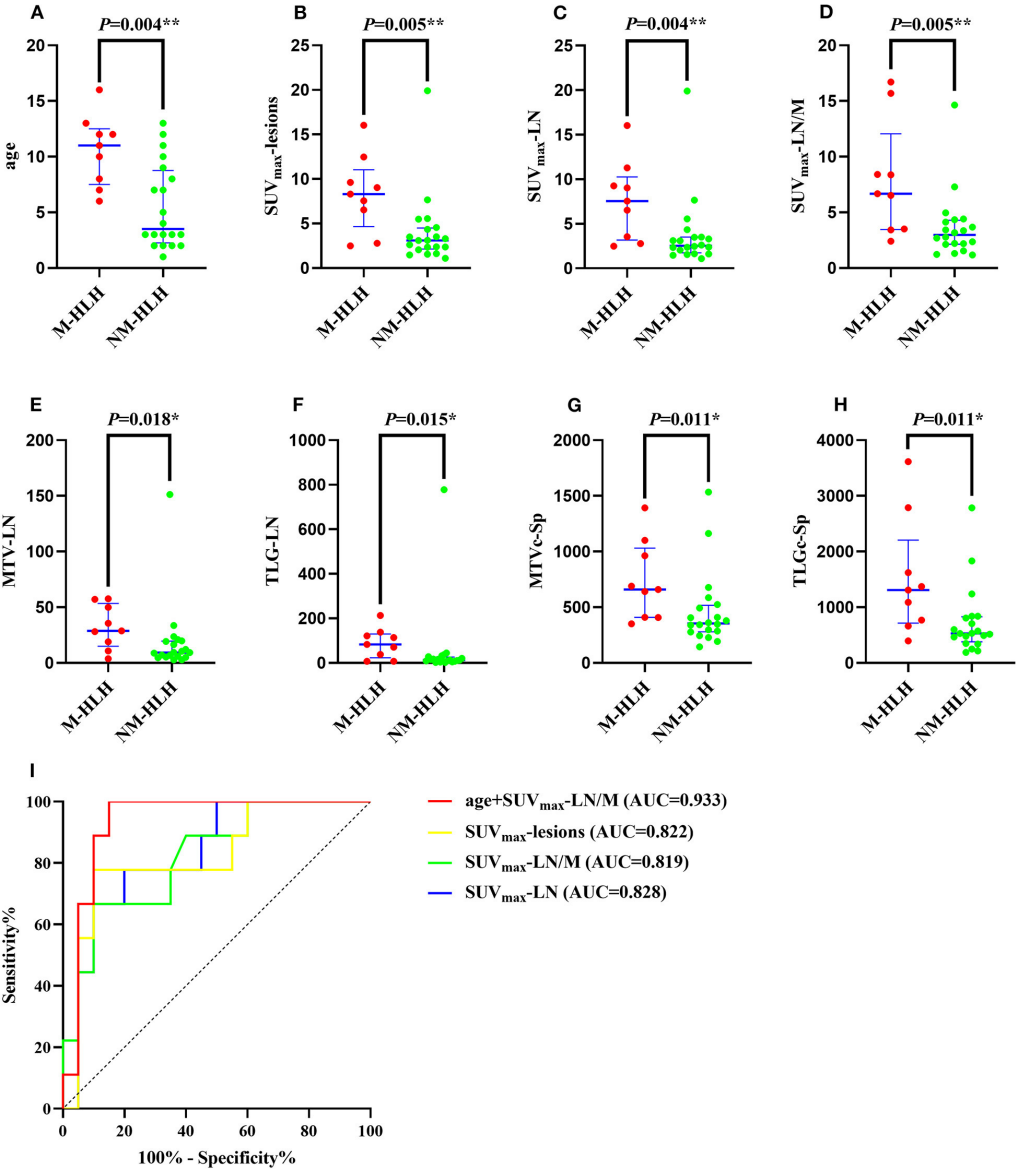
Diagnostic ability of metabolic and volumetric parameters of ^18^F-FDG PET/CT in differentiating M-HLH from NM-HLH. The age **(A)**, SUV_max_-lesions **(B)**, SUV_max_-LN **(C)**, SUV_max_-LN/M **(D)**, MTV-LN **(E)**, TLG-LN **(F)**, MTVc-Sp **(G)**, TLGc-Sp **(H)** were significantly higher in M-HLH than NM-HLH (*P* = 0.004, 0.005, 0.004, 0.005, 0.018, 0.015, 0.011, 0.011, respectively). Receiver operating characteristic curve indicated that the combination of age and SUV_max_-LN/M had the best diagnostic performance in differentiating M-HLH from NM-HLH (**I**, red line). Besides, SUV_max_-lesions was better than SUV_max_-LN and SUV_max_-LN/M, with higher sensitivity and accuracy (**I**, yellow line). HLH, hemophagocytic lymphohistiocytosis; EBV, Epstein-Barr virus; M-HLH, EBV-induced malignancy-associated HLH; NM-HLH, EBV-induced non-malignancy-associated HLH; SUV_max_, maximum standard uptake value; MTV, metabolic tumor volume; TLG, total lesion glycolysis; Sp, spleen; LN, lymph nodes; M, mediastinum; c, corrected; AUC, area under the curve. *P < 0.05, **P < 0.01.

Then the quantitative and semi-quantitative analysis were inducted, and the level of age, SUV_max_-lesions, SUV_max_-LN, SUV_max_-LN/M, MTV-LN, TLG-LN, MTVc-Sp and TLGc-Sp were significantly higher in M-HLH than in NM-HLH (*P* = 0.004, 0.005, 0.004, 0.005, 0.018, 0.015, 0.011, 0.011, respectively, Table 1; Figures 4A–H). However, there were no significant difference of the laboratory parameters and the other PET/CT findings between the two groups (Table 1).

The ROC curve analysis showed that the age, SUV_max_-lesions, SUV_max_-LN, SUV_max_-LN/M, MTV-LN, TLG-LN, MTVc-Sp and TLGc-Sp were all able to differentiate M-HLH from NM-HLH with AUC of 0.833, 0.822, 0.828, 0.819, 0.778, 0.783, 0.794, 0.794, respectively (Table 2). SUV_max_-LN/M (cut-off = 5.74) showed better performance than other parameters of lymph nodes and spleen, with high specificity (90%), low sensitivity (66.7%), and median accuracy (79.3%). Comparing to SUV_max_-LN/M, SUV_max_-lesions had higher sensitivity and accuracy, with the involving of the metabolic parameters of extranodal lesions (cut-off = 6.04, specificity = 90%, sensitivity = 77.8%, accuracy = 86.2%). The addition of combining variables might improve the diagnostic performance, with logistic regression method. Due to the linear correlation among the above PET parameters (variance inflation factor > 5), the age was combined with each PET parameter. Finally, combining the age with SUV_max_-LN/M showed the best diagnostic efficiency, with increased specificity of 85% and sensitivity of 100% (AUC = 0.933) (Table 2; Figure 4I).

**Table 2:** Diagnostic ability of metabolic and volumetric parameters of ^18^F-FDG PET/CT in differentiating M-HLH from NM-HLH.

**Parameters**	**Cut-off values**	**Sensitivity (%)**	**Specificity (%)**	**Accuracy (%)**	**AUC (95%CI)**
age	5.5	100.0	60.0	100.0	0.833 (0.688–0.979)
SUV_max_-lesions	6.04	77.8	90.0	86.2	0.822 (0.648–0.996)
SUV_max_-LN	6.04	66.7	90.0	58.6	0.828 (0.670–0.985)
SUV_max_-LN/M	5.74	66.7	90.0	79.3	0.819 (0.656–0.983)
MTV- LN	25.82	66.7	90.0	79.3	0.778 (0.575–0.981)
TLG-LN	37.33	77.8	90.0	86.2	0.783 (0.567–1.000)
MTVc-Sp	407.15	88.9	65.0	93.1	0.794 (0.630–0.959)
TLGc-Sp	630.01	88.9	65.0	93.1	0.794 (0.614–0.975)
Age + SUV_max_-lesions	/	100.0	85.0	/	0.911 (0.802–1.000)
Age + SUV_max_-LN	/	100.0	80.0	/	0.906 (0.796–1.000)
Age + SUV_max_-LN/M	/	100.0	85.0	/	0.933 (0.838–1.000)
Age + TLG-LN	/	66.7	85.0	/	0.839 (0.693–0.985)
Age + TLGc-Sp	/	77.8	90.0	/	0.883 (0.760–1.000)

*AUC, area under the curve; CI, confidence interval; HLH, hemophagocytic lymphohistiocytosis; EBV, Epstein-Barr virus; M-HLH, EBV-induced malignancy-associated HLH; NM-HLH, EBV-induced non-malignancy-associated HLH; Sp, spleen; LN, lymph nodes; M, mediastinum; SUV_max_, maximum standard uptake value; MTV, metabolic tumor volume; TLG, total lesion glycolysis; c, corrected*.

### Correlation Analysis Between ^18^F-FDG PET/CT Parameters and HLH Related Laboratory Parameters

In this study, the metabolic parameters of spleen and bone marrow correlated with the laboratory parameters related to HLH (Table 3, other parameters were not listed). SUV_max_-SP showed a low positive correlation with serum ferritin, TG, IFN-γ, IL-6 (*r* = 0.432, *P* = 0.019; *r* = 0.374, *P* = 0.046; *r* = 0.492, *P* = 0.007; *r* = 0.452, *P* = 0.014). SUV_max_-SP/M had a low to moderate positive correlation with TG, sCD25 and IL-6 (*r* = 0.370, *P* = 0.048; *r* = 0.374, *P* = 0.046; *r* = 0.514, *P* = 0.004). SUV_max_-BM had a moderate positive correlation with IFN-γ (*r* = 0.515, *P* = 0.004). SUV_max_-BM/M had a low negative correlation with Hb (*r* = −0.412, *P* = 0.026).

**Table 3:** Correlation analysis between ^18^F-FDG PET/CT parameters and HLH related laboratory parameters.

**Laboratory parameters**	**SUV_**max**_-SP**	**SUV_**max**_-SP/M**	**SUV_**max**_-BM**	**SUV_**max**_-BM/M**
Hb (g/L)	−0.141 (0.466)	−0.287 (0.131)	−0.300 (0.114)	−0.412 (0.026);*
Serum ferritin (ng/mL)	0.432 (0.019);*	0.342 (0.069)	0.235 (0.220)	0.118 (0.541)
TG (mmol/L)	0.374 (0.046);*	0.370 (0.048);*	−0.047 (0.810)	−0.087 (0.654)
sCD25 (pg/mL)	0.367 (0.050)	0.374 (0.046);*	0.199 (0.300)	0.126 (0.515)
IFN-γ (pg/mL)	0.492 (0.007)**	0.330 (0.080)	0.515 (0.004)**	0.340 (0.071)
IL-6 (pg/mL)	0.452 (0.014);*	0.514 (0.004)**	0.272 (0.154)	0.268 (0.160)

*HLH, hemophagocytic lymphohistiocytosis; Hb, hemoglobin; TG, triglyceride; sCD25, soluble CD25; IFN-γ, interferon-γ; IL: interleukin; SUV_max_, maximum standard uptake value; Sp, spleen; BM, bone marrow; M, mediastinum*.

* *P < 0.05*,

** *P < 0.01*.

### Prognostic Analysis

After a median follow-up of 88 weeks (range 3–156 weeks), 4 (13.8%) patients died, including two died of severe infection and multiple organs failure, one died of liver failure and disseminated intravascular coagulation before transplantation, and one died of severe infection after transplantation.

The treatment response of patients was evaluated at the 2nd, 4th, 6th, and 8th week (25, 24, 23 and 22 patients, respectively). The study found that the older age, increased TLGc-Sp, SUV_max_-BM, SUV_max_-LN, SUV_max_-lesions, and the existence of serous effusion were related to the poorer treatment response at the 2nd week (not reaching partial remission) (*P* = 0.03, 0.048, 0.007, 0.031, 0.014, 0.036, respectively). Older age, increased SUV_max_-LN, SUV_max_-lesions, and the existence of serous effusion were related to the poorer treatment response at the 4th week (*P* = 0.003, 0.047, 0.001, 0.047, respectively). The treatment response of the 6th and 8th week were not analyzed as there were few patients reaching partial remission (three and four patients, respectively).

**Figure 5:**
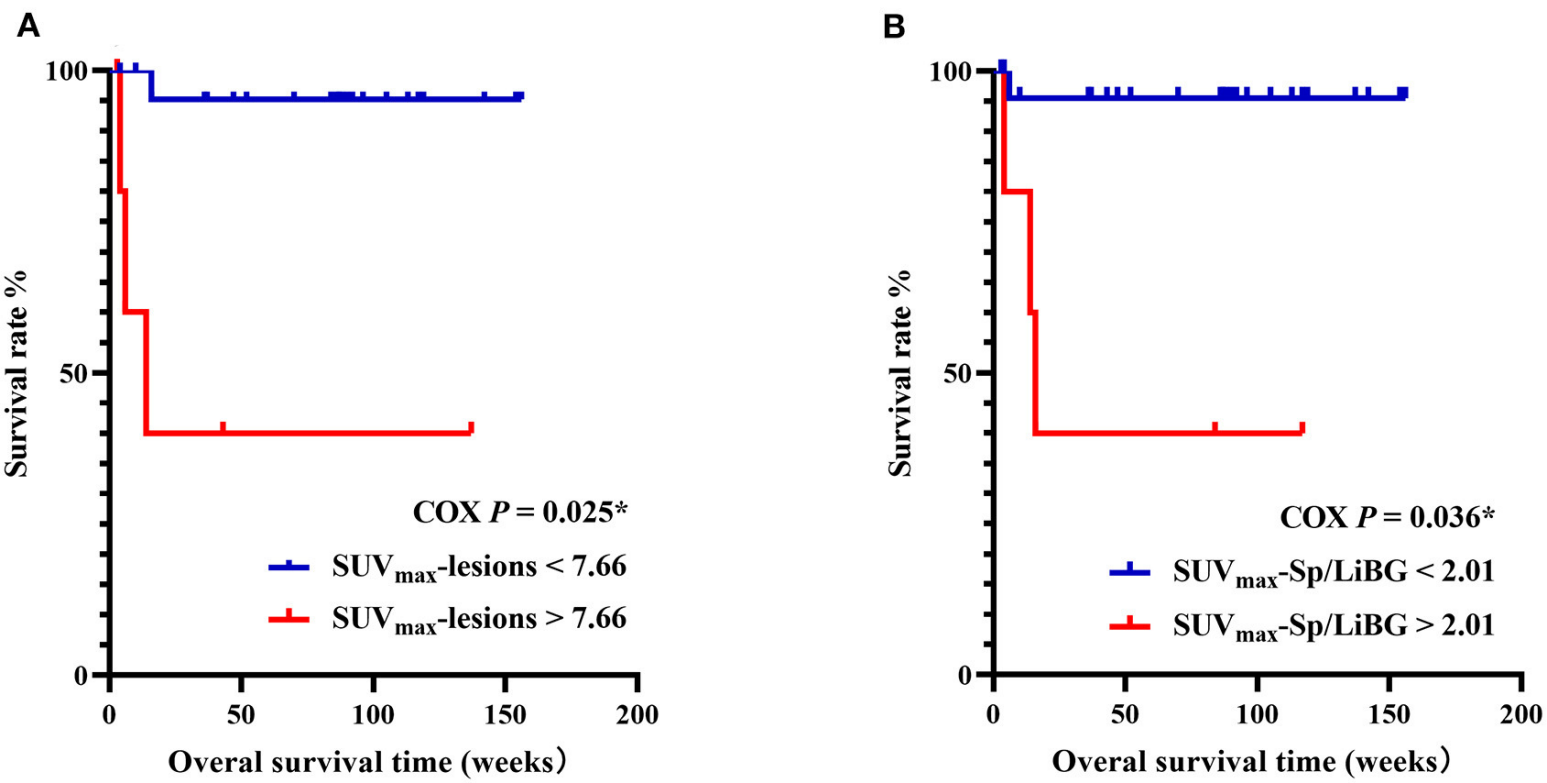
Kaplan–Meier survival curves of pediatric HLH with EBV infection, with SUV_max_-lesions **(A)**, SUV_max_-Sp/LiBG **(B)**. In multivariate analysis, SUV_max_-lesions > 7.66 and SUV_max_-Sp/LiBG > 2.01 were independent prognostic factors for overall survival (*P* = 0.025, *P* = 0.036, respectively). HLH, hemophagocytic lymphohistiocytosis; EBV, Epstein-Barr virus; SUV_max_, maximum standard uptake value; Sp, spleen; LiBG, liver background. * *P* < 0.05.

In the prediction of OS, the univariate analysis showed that the elevated metabolic parameters of the spleen, lymph nodes, and lesions, including SUV_max_-lesions, SUV_max_-LN, SUV_max_-LN/LiBG, SUV_max_-Sp, SUV_max_-Sp/M, SUV_max_-Sp/LiBG, and the level of IL-6, were prognostic factors (*P* < 0.001, *P* = 0.011, 0.019, 0.005, 0.016, 0.001, 0.018, respectively, Table 4). As the linear correlation among the metabolic parameters of the spleen or of the lymph nodes, only the parameters with the smallest *P* value were enrolled in multivariate analysis. Besides, the previous studies had reported that malignancy was a prognostic factor. Therefore, five parameters including malignancy, IL-6, SUV_max_-lesions, SUV_max_-Sp/LiBG, and SUV_max_-LN were analyzed. Finally, the multivariate analysis showed that only SUV_max_-lesions and SUV_max_-Sp/LiBG were independent prognostic factors (cut-off = 7.66, *P* = 0.025; cut-off = 2.01*, P* = 0.036) (Table 4; Figure 5).

**Table 4:** Univariate and multivariate analysis for overall survival in pediatric HLH with EBV infection.

**Variables**	**Univariate analysis**		**Multivariate analysis**	
	**X^**2**^**	***P*** **-value**	**HR (95%CI)**	***P*** **-value**
Malignancy (yes)	5.157	0.023	-	-
IL-6 (>26.5 pg/mL)	5.645	0.018^#^	-	-
SUV_max_-lesions (>7.66)	12.950	<0.001**	20.336 (1.460–283.191)	0.025;*
SUV_max_-LN (>3.31)	6.440	0.011^#^	-	-
SUV_max_-LN/LiBG (>2.62)	5.499	0.019^#^	/	/
SUV_max_-Sp (>3.13)	7.733	0.005^#^	/	/
SUV_max_-Sp/M (>2.96)	5.826	0.016^#^	-	-
SUV_max_-Sp/LiBG (>2.01)	10.619	0.001**	15.136 (1.187–192.952)	0.036;*

*HLH, hemophagocytic lymphohistiocytosis; EBV, Epstein-Barr virus; IL: interleukin; Sp, spleen; LN, lymph nodes; LiBG, liver background; M, mediastinum; SUV_max_, maximum standard uptake value*.

# *P < 0.02*,

* *P < 0.05*,

** *P < 0.01*.

## Discussion

It is quite important to distinguish M-HLH from NM-HLH, and to find other prognostic factors for pediatric HLH with EBV infection. For children with M-HLH and poor prognosis, it's necessary to take more active and earlier treatments, such as chemotherapy and hematopoietic stem cell transplant, which might improve the prognosis (6, 20). The present study found that SUV_max_-lesions, SUV_max_-LN/M, and the union of age and SUV_max_-LN/M had better diagnostic performance in differentiating M-HLH from NM-HLH. Besides, the presence of extranodal hypermetabolic lesions in multiple organs indicated the M-HLH. Moreover, ^18^F-FDG PET/CT parameters of the spleen and bone marrow correlated with some parameters related to the activity of HLH. The multivariate analysis showed that the SUV_max_-lesions > 7.66 and SUV_max_-Sp/LiBG > 2.01 were independent prognostic factors for OS.

There are no characteristic findings on ^18^F-FDG PET/CT in pediatric HLH with EBV infection. And the main purpose of using ^18^F-FDG PET/CT is to find neoplastic lesions in lymph nodes or extranodal organs and to guide biopsies (6). The general findings usually indicate high inflammatory status, including hepatomegaly and splenomegaly with elevated FDG uptake, diffuse elevated FDG uptake in the bone marrow, and serous effusion (21). In our study, there were also multiple lymphadenomegaly and extranodal lesions with increased FDG uptake except for the above findings. The extranodal involved organs included spleen, liver, bone marrow, brain, lung, intestine, kidney, adrenal gland, skin, nasal mucosa, and muscle, etc.

In the visual differential diagnosis in pediatric HLH with EBV infection, this study found that lymphoma-like presentation, including multiple enlarged lymph nodes with obviously increased FDG uptake, local mass of fused lymph nodes, and/or extronodal lesions especially in multiple organs, could indicate the M-HLH. However, enlarged lymph nodes are common in NM-HLH patients, such as infectious mononucleosis and LPD grade 1–2 (22, 23). Therefore, it is important to consider the size, metabolism, and anatomical changes of lymph nodes comprehensively. On the contrary, some kinds of lymphoma do not show enlarged lymph nodes, such as EBV associated extranodal NK/T cell lymphoma, mainly affecting extranodal tissue, which is common in Asia (24). A previous study reported that hypermetabolic lesions in extranodal organs, such as liver, spleen and bone marrow, often indicated the involvement of HL and invasive NHL (25). Hence, the presence of extranodal hypermetabolic lesions, especially in multiple extranodal organs, was of great significance in the diagnosis of M-HLH. And it was consistent with previous studies, which suggested single extranodal organ involvement sometimes indicated NM-HLH (23, 26–28).

The present study found that semi-quantitative measurement of metabolic parameters of lymph nodes and extranodal organs might be helpful in distinguishing M-HLH from NM-HLH. It's reported that the FDG uptake of lymph nodes is related with invasiveness of the disease (29). Okano et al. found that increased SUV_max_-LN and SUV_max_-lesions indicated the lymphoma-associated HLH with cut-off value of 3.3, 5.5, respectively (21). In the study, the level of FDG uptake of the lymph nodes and extranodal lesions, including SUV_max_-LN, SUV_max_-LN/M, MTV-LN, TLG-LN, and SUVmax-lesions, could help determine M-HLH. The best cut-off value of SUV_max_-LN and SUV_max_-lesions were both 6.04, which were higher than in adults, and it might be resulted from the physiological increased uptake of cervical lymph nodes in children (30). Comparing to SUV_max_-LN and SUV_max_-LN/M, the study showed that SUV_max_-lesions had similar AUC and higher sensitivity and accuracy in detecting M-HLH, which indicated that the addition of the metabolic information of extranodal lesions could help finding the M-HLH better. In addition, it's reported that elder children are more likely to develop M-HLH with EBV infection (28). So age was combined with the above metabolic parameters separately in the differentiation analysis in our study. And we found that the combination of age and SUV_max_-LN/M performed best. A study reported that some laboratory parameters, such as fibrinogen <1.5g/L, PLT <40g/L and LDH > 100U/L, were helpful in finding M-HLH (31). In our study, however, there was no significant difference in laboratory results between M-HLH and NM-HLH.

Due to the diversity and heterogeneity of pathology in EBV-associated disease, varying from LPD grade 1–3 to lymphoma, ^18^F-FDG PET/CT is recommend to find the possible neoplastic lesions and to determine the most appropriate biopsy site according to the metabolic and anatomic information (6). However, it's important for physicians to note the following tips in using PET/CT scans. Firstly, physicians should adequately consider the radiation dose for children, identify those who really need the scan by preliminary screening examination, such as ultrasound. Moreover, physicians should be aware of the limitations of ^18^F-FDG PET/CT, for example, false positive conditions in the Waldeyer's ring and gastrointestinal tract, and false negative conditions in mantle cell lymphoma and peripheral T-cell lymphoma (32). Furthermore, it's important to know that pathological biopsy is still the gold standard in differential diagnosis. In the study, one patient with T-cell NHL demonstrated as non-malignancy disease on PET/CT, only showing slightly increased FDG uptake in bone marrow. Besides, one NM-HLH patient mimicked the lymphoma on PET/CT, with multiple enlarged and hypermetabolic lymph nodes, and extranodal lesions in multiple organs (liver, bone marrow and lungs), which finally proved to be LPD grade 2.

The present study indicated that FDG uptake of the spleen and bone marrow might correlate with the inflammation of the body and the activity of HLH (33). Some laboratory parameters, including serum ferritin, TG, sCD25, IFN-γ, IL-6, IL-10 and TNF-α, could reflect the level of “inflammatory storm, which lead to the “sepsis-like symptoms” and organs injuries in HLH (34–36). It's reported that IFN-γ plays an essential role in the procedure, promoting the activation of mononuclear macrophages and resulting in hemophagocytosis (13). Yang et al. found that IL-6 seemed to play a similar but weaker role in causing organ damage than IFN-γ (34). In this study, the level of FDG uptake of the spleen positively correlated with IFN-γ and IL-6, which was consistent with previous studies. Besides, the metabolism of the bone marrow could reflect the hematopoiesis in it, because of its negative correlation with hemoglobin.

^18^F-FDG PET/CT parameters could predict the treatment response and OS in pediatric HLH with EBV infection, which were rarely studied in the previous studies. The present study showed that older age, higher SUV_max_-LN, and SUV_max_-lesions indicated worse therapeutic effect at early treatment evaluation at the 2nd and 4th week, for they were related to M-HLH. And the higher FDG uptake of the spleen and bone marrow might indicated worse therapeutic effect at the 2nd week, for they were related to higher inflammatory status.

Our study found that SUV_max_-Sp/LiBG and SUV_max_-lesions were independent prognostic factors in HLH with EBV infection children. The cut-off value of SUV_max_-Sp/LiBG (2.01) was higher than Kim's study in adult (1.19) (37). Kim et al. also suggested that elevated SUV_max_-BM, SUV_max_-Sp, SUV_max_-BM/LiBG were related to poorer prognosis (37). Besides, Zheng et al. reported that treatment strategy, serum fibrinogen and SUV_max_-Sp/M were independent prognostic factors (14). The elevated FDG uptake of spleen is reported to be resulted from the activation of T cells and macrophages stimulated by IFN-γ (38). In other disease, Xia et al. retrospectively analyzed 141 patients with extranodal NK/T lymphoma and found that SUVmax-lesions ≥ 9.65 was associated with poor prognosis (39).

In the study, the univariate analysis also showed that the metabolic parameters of lymph nodes and IL-6 were related to OS. The baseline SUV_max_, TLG and MTV of lymph nodes were related to OS in lymphoma, including HL, diffuse Large B-cell lymphoma, follicular lymphoma, peripheral T lymphocyte lymphoma, extranodal NK/T lymphoma, etc. (11, 40). Besides, elevated IL-6 indicates strong “inflammatory storm,” suggesting the internal environment disturbance and a high mortality rate. Lu et al. found that IL-6 ≥ 18.59 pg/mL was one of the independent prognostic factors in 155 adults with HLH (41). Chen et al. found that IL-6 and IL-10 were prognostic factors in children with CAEBV (9). In the study, we did not find the relationship between volumetric parameters of lymph nodes and prognosis, which might because of the small sample size.

There were some limitations in this retrospective study. Firstly, due to the insufficient sample size, there were no verification group. Secondly, despite using pathology as a standard reference, differentiation between different pathologic types remains challenging. In the future, studies with large sample size in multicenter are needed to evaluate the value of ^18^F-FDG PET/CT in pediatric HLH with EBV infection.

## Conclusion

In conclusion, the study finds that ^18^F-FDG PET/CT plays a certain role in pediatric HLH with EBV infection. M-HLH should be considered when SUV_max_-lesions > 6.04, SUV_max_-LN/M > 5.74 and the presence of extranodal hypermetabolic lesions in multiple organs. The FDG uptake of the spleen and bone marrow correlates with the activity of HLH. SUV_max_-lesions > 7.66 and SUV_max_-Sp/LiBG > 2.01 are independent prognostic factors for OS in pediatric HLH with EBV infection.

## Data Availability Statement

The original contributions presented in the study are included in the article, further inquiries can be directed to the corresponding authors.

## Author Contributions

XL and AW: conception and design of the study, protocol development, analysis, interpretation of data, and drafting the article. XY and JL: analysis and interpretation of data and revision of the article. SL: data collection. YK and WW: formal analysis. JY and RZ: project administration. All authors contributed to the article and approved the submitted version.

## Funding

This work was supported by the National Natural Science Foundation of China (Nos. 81771860 and 81971642), Beijing Natural Science Foundation (No. 7192041), National Key Research and Development Plan (No: 2020YFC0122000), National Science and Technology Key Projects (No. 2017ZX09304029001) and the Pediatric Medical Coordinated Development Centre of Beijing Municipal Administration of Hospitals (No. XTZD20180202).

## Conflict of Interest

The authors declare that the research was conducted in the absence of any commercial or financial relationships that could be construed as a potential conflict of interest.

## Publisher's Note

All claims expressed in this article are solely those of the authors and do not necessarily represent those of their affiliated organizations, or those of the publisher, the editors and the reviewers. Any product that may be evaluated in this article, or claim that may be made by its manufacturer, is not guaranteed or endorsed by the publisher.

## Abbreviations

M-HLH: EBV-induced malignancy-associated HLH
NM-HLH: EBV-induced non-malignancy-associated HLH
EBV-HLH: HLH driven by pure EBV infection
Li: liver
Sp: spleen
BM: bone marrow
LN: lymph nodes
LiBG: liver background
M: mediastinum.
